# Trait mindfulness in early pregnancy and adverse perinatal outcomes: a prospective cohort study

**DOI:** 10.1186/s12884-025-07194-y

**Published:** 2025-01-24

**Authors:** Audra C. Fain, Tess E. K. Cersonsky, Margaret H. Bublitz, Adam K. Lewkowitz, Erika F. Werner, Emily S. Miller, Nina K. Ayala

**Affiliations:** 1 Department of Obstetrics and Gynecology, David Geffen School of Medicine UCLA, 10833 Le Conte Ave, Los Angeles, CA CHS B2-049 USA; 2 Department of Obstetrics and Gynecology, Icahn School of Medicine Mount Sinai, New York, NY USA; 3 Department of Psychiatry and Human Behavior, Alpert Medical School of Brown University, Providence, RI USA; 4 Department of Medicine, Alpert Medical School of Brown University, Providence, RI USA; 5 Division of Maternal Fetal Medicine, Women and Infants Hospital of Rhode Island, Providence, RI USA; 6 Department of Obstetrics and Gynecology, Alpert Medical School of Brown University, Providence, RI USA; 7 Department of Obstetrics and Gynecology, Tufts University School of Medicine, Boston, MA USA

**Keywords:** Trait mindfulness, Pregnancy, Maternal morbidity, Adverse perinatal outcomes

## Abstract

**Background:**

Mindfulness centered therapy has been shown to improve perinatal mental health outcomes. There is emerging evidence that mindfulness training (MT) can also be harnessed to improve somatic outcomes. Yet, little is known about which perinatal populations might benefit the most from mindfulness training interventions. We aimed to evaluate the association between trait mindfulness and adverse pregnancy outcomes.

**Methods:**

This is a planned secondary analysis of a prospective cohort study of nulliparous participants recruited between May 2019 and February 2022 from a single, high volume tertiary care center. Participants completed the validated Mindfulness and Attentive Awareness Scale prior to 20 weeks gestation. Trained research staff abstracted pregnancy and delivery data. The primary outcome was unplanned cesarean delivery (CD). Secondary outcomes included gestational diabetes, hypertensive disorders of pregnancy and a neonatal morbidity composite. We examined outcomes by mindfulness quartile (Q), adjusting for covariates determined a priori.

**Results:**

Of the 281 participants with full outcome data, 47.9% experienced one or more of the adverse perinatal outcomes and the median trait mindfulness score was 4.6 (IQR 3.9–5.3). After adjusting for potential confounders, there were significantly lower rates of CD rates in those in Q2 and Q3 compared to Q4 (adjusted odds ratio [aOR] Q2 0.42, 95% confidence interval [CI] 0.20 – 0.87, Q3 aOR 0.23, 95% CI 0.10–0.51). There were no differences in rates of gestational diabetes, hypertension or composite neonatal outcomes by trait mindfulness quartile.

**Conclusions:**

In this prospective cohort of nulliparous people, those with trait mindfulness in the 2nd and 3rd quartiles had lower rates of CD. Given prior literature suggesting active MT decreases adverse outcomes, there may be a component of the active practice of mindfulness, rather than trait mindfulness levels, associated with improved outcomes.

## Introduction

The role of mindfulness, defined as a non-judgmental, open awareness of the present moment [1–3], in health promotion has garnered significant attention over the last couple decades. Mindfulness is now recognized as both a personal characteristic (“trait”) that is stable without targeted intervention [4, 5] but also a skill that can be improved and trained with practice [1, 3]. Much of the existing literature focuses on harnessing mindfulness interventions to improve mental health; much less is known about the role of trait mindfulness in health promotion and adverse outcomes, especially in pregnancy.

In non-pregnant populations, studies have shown that mindfulness-based interventions have beneficial impacts on cardiovascular response to stressors and blood sugar regulation among people living with diabetes [6, 7]. Within pregnancy, mindfulness training in pregnancy has been shown to reduce symptoms of anxiety and depression in the perinatal period [8–14], promise in reducing blood pressures among those at risk for hypertensive disorders of pregnancy [15, 16], improvements in neonatal and childhood neurodevelopment [17, 18] and decreased proportion of small placentas at time of delivery [19]. More recently, mindfulness-based training programs have also been shown to improve psychosocial and psychological outcomes for patients [20, 21], reduce anxiety and depression in both pregnant women and their partners when both parties attended the course [22], and were more effective than Lamaze courses at reducing perceived stress [23]. However, little existing research exists on the potential relationship between trait mindfulness and obstetric outcomes. If trait mindfulness is associated with obstetric outcomes, it may provide a basis for effectively targeting populations for whom mindfulness-based interventions would provide the greatest benefit.

Thus, we created a prospective cohort study to evaluate trait mindfulness in early pregnancy and its relationship with perinatal outcomes. Given literature showing that mindfulness-based interventions have beneficial impacts on mental health and the known association between poor mental health and adverse pregnancy outcomes, we hypothesized that higher early pregnancy mindfulness would be associated with reduction in adverse perinatal outcomes (APOs), including unscheduled CD, gestational diabetes mellitus (GDM), and hypertensive disorders of pregnancy (HDP).

## Methods

This is a planned secondary analysis of a prospective cohort study of participants from a single tertiary care center recruited from the outpatient care sites between May 2019 and February 2022 [24]. Recruitment paused between April and October 2020 given institutional guidelines during the COVID-19 pandemic. Inclusion criteria included nulliparous pregnant participants less than 20 weeks gestation at the time of who spoke English or Spanish, had a singleton fetus, and planned to deliver at the same medical center. Exclusion criteria included fetal major congenital anomalies given the potential impact on mode of delivery, a key study outcome as well as presence of pre-gestational diabetes or hypertension. Prior to participant recruitment, the institutional review board approved this protocol. All patients at the tertiary care center were screened during prenatal appointments during the enrollment period, and any participant who met eligibility criteria was approached and offered enrollment. Details on sample size and power calculations can be found in the publication by Ayala et al. [24]. Ethical considerations included short nature of the participant surveys to ensure no delay in prenatal visits or care, and recruitment was done by research personnel unrelated to patient care to minimize any pressure to enroll.

Participants completed a baseline questionnaire at enrollment including demographic questions and the Mindfulness Attention and Awareness Scale (MAAS). Pregnancy and delivery information including gestational age at delivery, induction of labor, mode of delivery (spontaneous vaginal delivery, vacuum assisted vaginal delivery, forceps assisted vaginal delivery or cesarean delivery), neonatal birthweight, Apgar scores, Neonatal intensive care unit (NICU) admission, and presence of a congenital anomaly were abstracted from the maternal and neonatal electronic health records following hospital discharge. Trained research staff completed the chart abstraction. Random selection of 5% of charts were double entered to ensure data collection accuracy. The research staff who completed data abstraction were blinded to baseline questionnaire results.

Mindfulness was assessed using the MAAS, a 15-item scale with answers scored on a 6-point Likert scale with 1 being “almost always” and 6 being “almost never” [25]. Questions center on a participant's ability to be aware of the present moment (for example, “I do jobs or tasks automatically, without being aware of what I'm doing”). The scale is scored based on the mean response from the 15 questions; possible scores range from 1–6, with a higher score indicating a higher mindfulness level. Prior studies have demonstrated good internal validity, with Cronbach’s alpha coefficients ranging from 0.80–0.87 [25].

The primary outcome was unscheduled CD, which was selected given the established relationship between mindfulness, stress parameters, and cesarean delivery. Secondary outcomes included GDM, HDP, and a neonatal morbidity composite. GDM was diagnosed according to the Carpenter-Coustan Criteria [26]. HDP included gestational hypertension, pre-eclampsia with and without severe features, eclampsia, and HELLP syndrome, defined using the American Congress of Obstetricians and Gynecologists (ACOG) criteria [27]. The composite neonatal morbidity variable was positive in the setting of any of the following outcomes: preterm birth (delivery at 20w0d-36w6d), NICU admission, umbilical artery pH < 7.0, 5-min Apgar score < 7, neonatal hyperbilirubinemia, hypoglycemia, and/or death.

Given this was a secondary analysis with a fixed sample size, we completed a post-hoc power analysis. Assuming the baseline rate of unplanned CD of 44% in quartile 4, we determined that we had 80% power to detect a 24 point decrease, 63% to detect a 20 point decrease, and 50% to detect a 16 point decrease.

R (version 4.2.3) was used for all statistical analyses. Maternal demographic and pregnancy characteristics were compared using Mann–Whitney and Chi-squared tests. Outcomes were assessed by mindfulness quartile using multivariable logistic regression controlling for confounders determined a priori. The referent group was defined as the highest mindfulness score quartile for all analyses, and multivariable analyses were adjusted for multiple comparisons using Hochberg’s Step-Up Procedure. A post-hoc test of trend was completed for outcomes that were significant in multivariable analysis to evaluate for a dose–response relationship between trait mindfulness and outcomes.

## Results

**Fig. 1:**
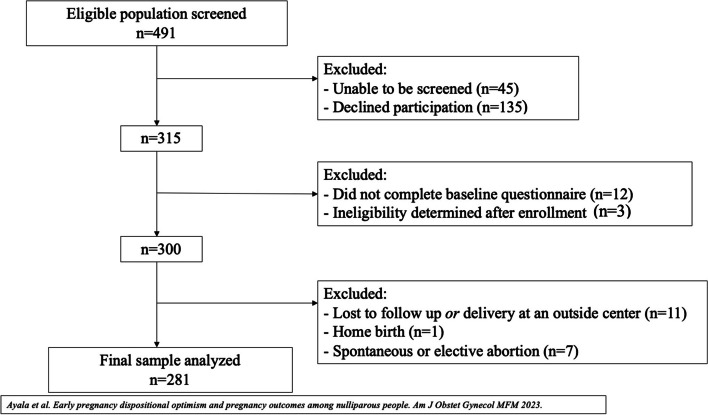
Study enrollment to analysis flow

All 281 participants with complete outcome data from the parent study were included (Fig. 1). The median MAAS score was 4.6 (IQR 3.9–5.3), and 47.9% had one or more APOs. Demographic characteristics including self-reported race/ethnicity and history of anxiety disorder differed significantly by mindfulness quartile (Table 1). There were no differences in participant age at enrollment, gestational age at enrollment, education, marital status, employment status, or history of depressive disorder by mindfulness quartile. In terms of pregnancy and labor characteristics, there was a significant difference in birthweight by mindfulness quartile, but no difference in gestational age at delivery, or induction of labor.

**Table 1:**
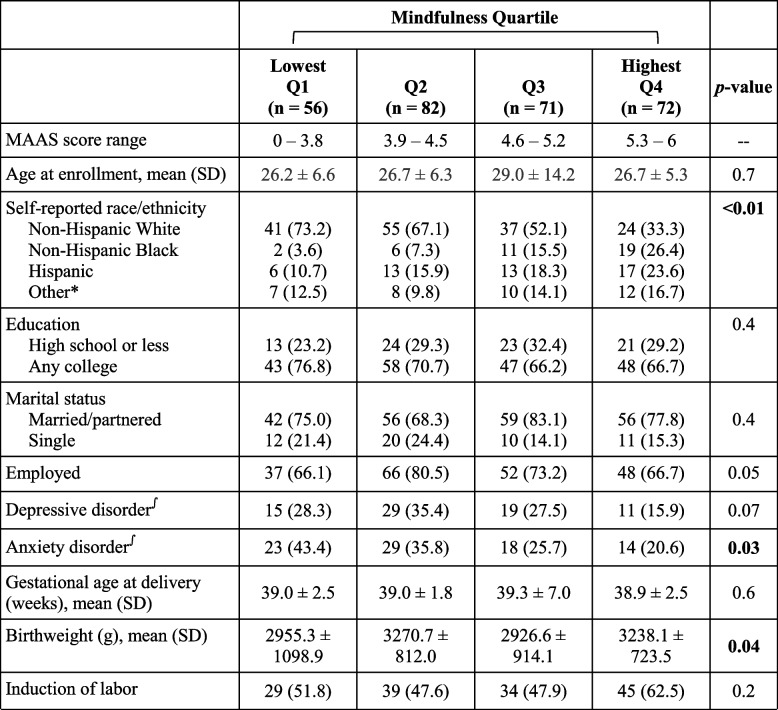
Demographic, pregnancy and delivery characteristics of parturients by mindfulness quartile in early pregnancy

Columns are n (%) unless otherwise indicated, significance at *p* < 0.05 (bold)

∫Participant self-report of prior or current diagnosis of depression and/or anxiety

*p-*value calculated between Q1 and Q4

After adjusting for confounders, participants in the second and third mindfulness quartiles had significantly lower odds of undergoing unplanned CD than participants in the highest mindfulness quartile (Q2 adjusted odds ratio [aOR] 0.42, 95% confidence interval [CI] 0.20–0.87, Q3 aOR 0.23, 95% CI 0.10–0.51) (Table 2). There was no difference in rates of unplanned CD when comparing Q1 with upper quartiles (aOR 0.51, 95% CI 0.23—1.11). In terms of secondary outcomes, there was no difference in odds of GDM, HDP or the neonatal morbidity composite among different mindfulness quartiles.

**Table 2:** Multivariable regression of adverse perinatal outcomes by mindfulness quartile

Mindfulness Quartile	Outcome^a^	Odds ratio (OR)^c^	Adjusted Odds Ratio (aOR)^d^
**Primary outcome: Unplanned cesarean delivery**			
Q1 (lowest)	16 (29.1)	0.47 (0.22—0.99)	0.51 (0.23—1.11)
Q2	24 (29.3)	0.48 (0.24—0.93)	0.42 (0.20—0.87)
Q3	15 (21.1)	0.29 (0.13—0.60)	0.23 (0.10—0.51)
Q4 (highest)	33 (45.8)	Referent	Referent
**Gestational diabetes**			
Q1 (lowest)	4 (7.1)	1.73 (0.01—9.08)	2.21 (0.44—12.5)
Q2	8 (9.8)	2.38 (0.66—11.2)	2.64 (0.68—13.3)
Q3	3 (4.2)	0.99 (0.18—5.49)	0.94 (0.16—5.38)
Q4 (highest)	3 (4.2)	Referent	Referent
**Hypertensive disorders of pregnancy**			
Q1 (lowest)	13 (23.2)	1.03 (0.44—2.36)	0.87 (0.35—2.12)
Q2	18 (22.0)	0.93 (0.43—2.02)	0.78 (0.35—1.74)
Q3	16 (22.5)	0.98 (0.44—2.17)	0.72 (0.30—1.68)
Q4 (highest)	18 (25.0)	Referent	Referent
**Composite neonatal morbidity**^**b**^			
Q1 (lowest)	19 (33.9)	0.97 (0.45—2.06)	1.02 (0.47—2.24)
Q2	22 (26.8)	0.73 (0.36—1.48)	0.82 (0.40—1.69)
Q3	22 (31.0)	0.86 (0.42—1.75)	0.82 (0.39—1.71)
Q4 (highest)	23 (31.9)	Referent	Referent

Quartile 4 (highest) is referent in all analyses

^a^Column is n (%)

^b^Neonatal morbidity composite: preterm birth, admission to the Neonatal Intensive Care unit (NICU), APGAR < 7 at 5 min, umbilical artery pH < 7.0, neonatal hypoglycemia, hyperbilirubinemia requiring treatment and neonatal death

^c^Odds of outcome (with 95% confidence interval [CI]) based on score on MAAS

^d^Odds of outcome (with 95% confidence interval [CI]) based on score on MAAS, adjusted for maternal age at enrollment, years of education, and self-reported diagnosis of depression or anxiety

## Discussion

In this secondary analysis of a prospective cohort study of pregnant people delivering for the first time, there were lower rates of CD among those in the 2nd and 3rd quartile of mindfulness compared to the highest quartile.

The significant aOR found for Q2 and Q3 but not for Q1 or Q4 deviates from a classic dose–response expectation. This finding suggests that moderate levels of mindfulness (as seen in Q2 and Q3) might strike an optimal balance—enough to foster a calm and focused state beneficial during labor, but not so much that it amplifies stress or over-attentiveness to negative experiences.

The non-significant finding for Q1 suggests that low mindfulness does not have a strong protective or harmful effect relative to the highest quartile, while moderate mindfulness (Q2 and Q3) provides a sweet spot where trait mindfulness might confer a protective benefit. In contrast, high mindfulness (Q4) might cross a threshold where the potential negative effects of heightened awareness begins to outweigh the benefits.

The majority of prior perinatal mindfulness studies have focused on mental health and patient reported outcomes related to childbirth experience, but there is emerging evidence that mindfulness may impact somatic outcomes as well [15, 16]. Prior studies that have assessed unexpected CD as an outcome have found that mindfulness training decreases rates of unplanned CD [28, 29]. Therefore, our data showing elevated CD rates among those with highest trait mindfulness were unanticipated. We posit that this may result from a trait-level heightened awareness of the self and surroundings without the intentional non-judgmental focus that is honed through mindfulness training paradigms [3, 30]. This unchecked awareness has the potential to lead to maladaptive responses, thereby increasing the risk for CD and adverse birth experiences.

This study found associations in the univariate analyses between trait mindfulness and race, anxiety, and infant birthweight (Table 1). There is limited research on how trait mindfulness varies across self-reported racial and ethnic groups in pregnant populations, although it has been found to be consistent across race and ethnicity in college cohorts [31]. There may be external factors unique to pregnancy uncaptured by this study that influence the relationship between race/ethnicity and trait mindfulness, which would be worth exploring in subsequent studies.

Results from this study highlight some challenges in measuring mindfulness. We utilized the MAAS for evaluating trait mindfulness, yet this may not have provided the nuanced discrimination of the different facets of mindfulness that would have been informative in putting our results into context. Other mindfulness scales such as the Five Facet Mindfulness Questionnaire [32], and the short-form Three Facet Mindfulness Questionnaire [33–35], evaluate different aspects of mindfulness including: observing, describing, acting with awareness, non-judging of inner experience and non-reactivity to inner experience. Future studies on trait mindfulness and perinatal outcomes should aim to utilize mindfulness scales that provide more robust information about the particular aspects of trait mindfulness that might alter somatic, mental health and patient reported outcomes in the perinatal period. Furthermore, trait mindfulness levels may be impacted by environmental or ecosocial factors, which should be explored in future studies.

The strengths of this study include the diverse participant populations and low lost-to-follow-up rate. By measuring mindfulness prior to 20 weeks gestation, we assessed trait mindfulness in early pregnancy rather than a mindfulness level affected by pregnancy complications or outcomes. By utilizing research staff for maternal and neonatal chart abstraction who were blinded to the MAAS survey results and cross-referencing abstraction for 5% of the charts, we minimized bias and inaccuracy in data collection.

However, our study contains several limitations. We did not assess mindfulness sequentially during pregnancy especially in the third-trimester or at delivery, so we cannot assess if naturally occurring changes in mindfulness throughout pregnancy are associated with APOs. However, without targeted intervention, mindfulness has been shown to be a stable characteristic [4, 5]. Thus, we would not expect to find systematic changes over pregnancy. We were underpowered to detect smaller effect sizes in outcomes, which means that clinically relevant differences in unplanned CD rates may not have reached statistical significance due to lack of precision around point estimates. Replicating these findings in a larger sample would be valuable moving forward. Furthermore, we did not collect patient centered outcomes related to pregnancy or birth experience, and thus have limited context with which to interpret our findings of highest CD rates among those with highest trait mindfulness. Furthermore, while we collected data on patient-reported histories of depressive or anxiety disorders, we did not review their charts for other psychiatric comorbidities—including PTSD, psychotic disorders, and personality disorders – which may be confounding factors related to mode of delivery. This limits the generalizability of our work to patients with a history of anxiety and depressive disorders but without the additional psychiatric comorbidities. We also did not record the mode of conception – including spontaneous, IUI, and IVF – which is significant as this can affect the rate of cesarean delivery.

An area our study did not assess but has potential for further research is additional psychosocial factors that may influence rates of unplanned CD. This includes preconceptions about recovery time from vaginal versus cesarean birth, complication rates, fear of labor pain, prior birth experiences – including traumatic prior vaginal or cesarean deliveries—and sense of control. These will be valuable to assess in further studies.

In conclusion, trait mindfulness was associated with differences in unscheduled CD, with those in the highest quartile at the highest risk. These findings underscore a complex, non-linear relationship between mindfulness and unplanned CD, where moderate levels might be most beneficial. Given prior literature suggesting mindfulness training reduces adverse outcomes both in pregnant and non-pregnant patients, there may be an element of active mindfulness practice that mediates adverse outcomes rather than trait mindfulness levels.

## Data Availability

The datasets generated and/or analyzed during the current study are not publicly available due Women and Infants IRB policy, but are available from the corresponding author on reasonable request.
